# Polarity-Driven Selective Adsorption of Quercetin on Kaolinite: An Integrated DFT and Monte Carlo Study

**DOI:** 10.3390/ma19020368

**Published:** 2026-01-16

**Authors:** Abdelilah Ayad, Achraf Harrou, Abdelouahad El Himri, Mohammed Benali, Abdelouassia Dira, Santiago Aparicio, Alberto Gutiérrez, Armand Soldera, Elkhadir Gharibi

**Affiliations:** 1 Laboratory of Applied Chemistry and Environment, Faculty of Sciences, Mohammed First University, BP 717, Oujda 60000, Morocco; harrou201@gmail.com (A.H.); elhimri.abdelouahad@gmail.com (A.E.H.); abdelouassia.dira@ump.ac.ma (A.D.); 2 Université de Technologie de Compiègne, Ecole Supérieure de Chimie Organique et Minérale, TIMR (Integrated Transformations of Renewable Matter), Centre de Recherches de Royallieu-CS, 60319-60203 Compiègne Cedex, France; m.benali@escom.fr; 3 Department of Chemistry, University of Burgos, 09001 Burgos, Spain; agvega@ubu.es; 4 International Research Centre in Critical Raw Materials-ICCRAM, University of Burgos, 09001 Burgos, Spain; 5 Department of Chemistry, Université de Sherbrooke, Sherbrooke, QC J1K 2R1, Canada

**Keywords:** clay, flavonoid, surface engineering, nanocarrier design, food, encapsulation

## Abstract

Quercetin’s therapeutic potential is limited by its poor water solubility and rapid degradation. Natural clay minerals such as kaolinite present sustainable platforms for drug delivery, yet the molecular mechanisms of drug encapsulation are not fully understood. Specifically, the role of kaolinite’s structural polarity, its hydrophilic aluminol (001) and hydrophobic siloxane (00-1) basal surfaces, in selective drug adsorption remains unexplored. This study combines Monte Carlo sampling and Density Functional Theory (DFT) to provide the first quantitative, atomistic comparison of quercetin adsorption on both kaolinite surfaces. The results demonstrate a pronounced polarity-driven selectivity. Strong, exothermic adsorption (−206.65 kJ mol^−1^) occurs on the hydrophilic (001) surface, stabilized by a network of five hydrogen bonds. In contrast, the hydrophobic (00-1) surface exhibits significantly weaker sorption (−147.16 kJ mol^−1^), dominated by van der Waals interactions. Charge-transfer analysis shows that the hydrophilic (001) surface exhibits a net charge transfer of −0.198 e, approximately 2.4 times greater than that of the hydrophobic (00-1) surface (−0.083 e), consistent with differential electron density maps and partial density of states. By linking hydrogen bonding and charge transfer to adsorption energy, these results elucidate how surface polarity dictates drug encapsulation. This work establishes a predictive framework for designing kaolinite-based nanocarriers with optimized stability, bioavailability, and controlled release, guiding the development of sustainable drug delivery systems. It is noted that this DFT study models adsorption at 0 K using periodic slab models in a vacuum.

## 1. Introduction

The controlled delivery of bioactive compounds remains a central challenge in pharmaceutical, nutraceutical, and cosmetic sciences. Many therapeutic agents suffer from poor aqueous solubility, low bioavailability, and rapid degradation, leading to insufficient therapeutic outcomes [1]. A prominent example is quercetin, a polyphenolic flavonoid with well-documented antioxidant, anti-inflammatory, and anticancer properties against cardiovascular diseases and various malignancies [2,3,4,5]. However, its clinical translation is severely hindered by a poor pharmacokinetic profile, characterized by extremely low water solubility (<10 μg mL^−1^), chemical instability, and oral bioavailability, often below 10% [6,7,8]. These limitations underscore the urgent need for advanced delivery platforms.

Natural clay minerals, such as kaolinite, halloysite, and montmorillonite, have emerged as promising green, low-cost carriers due to their abundance, biocompatibility, high surface area, and tunable surface chemistry [9,10,11]. Kaolinite (Al_2_Si_2_O_5_(OH)_4_) is particularly notable for its structural simplicity and distinctive interfacial chemistry. Its structure consists of alternating tetrahedral (SiO_4_) and octahedral (AlO_2_(OH)_4_) sheets, resulting in a pronounced intrinsic structural polarity with two chemically distinct basal surfaces: a hydrophilic, hydroxylated aluminol-terminated (001) surface and a hydrophobic siloxane-terminated (00-1) surface [12,13,14,15]. This polarity is complemented by pH-dependent edge sites (aluminol and silanol groups) that dominate surface charge and reactivity, contributing additional acidic/basic sites and hydration microstructures that significantly alter adsorption energetics [16,17,18]. This unique Janus-type character, underpins kaolinite’s diverse adsorption behaviors and relevance for drug delivery [19].

The intrinsic surface polarity dictates molecular adsorption mechanisms: the hydrophilic (001) surface promotes directional hydrogen-bonding, while the hydrophobic (00-1) surface engages primarily in non-directional van der Waals interactions [20,21]. Despite this fundamental difference, previous studies on quercetin have not explicitly addressed how surface polarity controls adsorption selectivity, strength, and orientation, leaving molecular-level mechanisms poorly understood and limiting rational nanocarrier design.

Previous computational (DFT, MD) and experimental research has identified hydrogen bonding and dispersion as dominant mechanisms in clay-molecule interactions [22,23,24,25], including studies on pharmaceuticals and polymers [26,27,28]. However, most studies have examined only one kaolinite surface, neglecting the interplay between basal and edge surfaces and risking incomplete conclusions [29,30]. Flavonoid adsorption introduces further complexity from pH-dependent speciation, metal chelation [31], and competitive hydration, often overlooked in simple vacuum models [32,33].

A surface-resolved, mechanistically grounded study is therefore essential to bridge phenomenological data with molecular-level insights. A direct quantitative comparison of quercetin adsorption on both basal planes is needed to test the hypothesized polarity-governed switch in binding mechanisms [34], requiring integrated configurational sampling and electronic-structure analysis.

This work reports the first integrated Monte Carlo (MC) and DFT study of quercetin adsorption on both pristine kaolinite basal surfaces. Our two-step computational workflow combines: (i) MC sampling to explore conformational and translational space, identifying thermodynamically favorable geometries, and (ii) DFT calculations with modern dispersion corrections to refine structures and quantify adsorption energetics, hydrogen-bond topologies, and electronic structure changes. To ensure physiological relevance, we consider quercetin’s pH-dependent protonation states and employ MD-derived potential of mean force calculations to evaluate free energy profiles under explicit hydration [35,36].

This integrated approach isolates the effects of intrinsic kaolinite surface polarity, revealing a polarity-driven transition in binding mechanisms and providing direct evidence of how hydrogen bonding and charge transfer govern adsorption strength and release dynamics. To our knowledge, this is the first such investigation on both basal faces of kaolinite, extending prior DFT studies on other adsorbates [37,38]. The resulting framework offers a predictive basis for designing sustainable, clay-based nanocarriers to enhance quercetin’s stability, bioavailability, and controlled release.

## 2. Computational Methods and Models

### 2.1. Quercetin and Kaolinite

**Figure 1:**
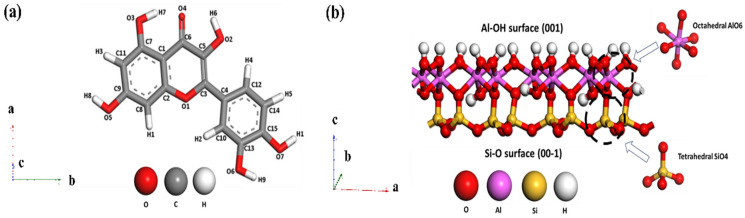
Chemical structure of (**a**) Quercetin (3,5,7,3′,4′-pentahydroxyflavone) and (**b**) Kaolinite.

Quercetin (C_15_H_10_O_7_, Figure 1a) is a flavonoid characterized by a complex hydrogen-bonding network. In its solid state, it forms nearly perpendicular dimers via intermolecular hydrogen bonds, creating a two-dimensional network interconnected by water molecules predominantly within the ab plane [39]. This compound crystallizes in a monoclinic system (space group P2_1_/a) [40], or alternatively in an orthorhombic system (space group Pn2_1_a) with unit cell parameters: *a* = 14.7998 Å, *b* = 11.2379 Å and *c* = 10.3512 Å, *α* = 90°, *β* = 90°, *γ* = 90 [41]. Its five hydroxyl groups and single carbonyl moiety facilitate extensive intra- and intermolecular hydrogen bonding [39]. Furthermore, quercetin demonstrates a specific affinity for aluminosilicate structures, having been shown to facilitate the precipitation of 1:1 aluminosilicates at 25 °C under aqueous conditions (pH 6.5–8.5), which are directly relevant to the natural formation environments of kaolinite [42].

Kaolinite (Figure 1b), with the structural formula Al_2_Si_2_O_5_(OH)_4_ and triclinic space group C1 [43], belongs to the triclinic system [44]. Due to its layered structure, kaolinite microparticles appear as hexagonal plates [45] with dominant, almost perfectly cleaved basal surfaces (001) and (00-1) [46]. The (001) surface is composed of OH groups bonded to Al atoms and is called the aluminol surface; the (00-1) surface is composed of oxygen atoms bonded to Si atoms and is called a siloxane [47].

The methodological approach combining DFT and MC for the molecular modeling of clay minerals, being a broader theoretical framework for adsorption energy calculations, allows the description of nanoscale energy variation within clay minerals, covering hydration, interaction, free and adsorption energy [48].

### 2.2. Density Functional Theory (DFT)

All spin-restricted Density Functional Theory (DFT) calculations were performed using the CASTEP [49] and Dmol3 [50] modules within the Materials Studio 2024 software. The exchange-correlation functional was treated with the Perdew–Burke–Ernzerhof generalized gradient approximation (PBE-GGA) [51]. Because adsorption at organic–mineral interfaces is governed by significant long-range noncovalent interactions, van der Waals (vdW) forces were explicitly included for using the Tkatchenko–Scheffler (TS) dispersion correction scheme (PBE + TS). This is critical because semi-local GGA functionals such as PBE, systematically underestimate adsorption energies when dispersion contributions are significant, particularly on siloxane-terminated surfaces. The TS approach incorporates environment-dependent dispersion coefficients derived from the electron density, enabling a more realistic description of heterogeneous inorganic-organic interfaces. The reliability of the PBE-TS method has been validated for organic molecule adsorption on oxides [52] and graphene-adsorbate systems, showing low errors and established [53]. The approach was benchmarked against reference data sets for polar mineral surfaces to ensure robustness [18,54]. This integrated approach, combining Monte Carlo (MC) and DFT, provides a comprehensive atomistic perspective, resolving energetics, charge distribution, and bond geometries. This detailed analysis of frontier orbitals and the use of PBE + TS methodology are critical for understanding the selective adsorption mechanisms of quercetin on kaolinite, providing a robust framework for rational nanocarrier design.

Geometry optimizations used the Broyden–Fletcher–Goldfarb–Shanno (BFGS) algorithm [55] with stringent convergence thresholds: energy < 1 × 10^−5^ eV/atom, max force < 0.03 eV/Å, max stress < 0.1 GPa, and max displacement < 2 × 10^−3^ Å [56]. A plane-wave kinetic energy cutoff of 450 eV was used, determined from convergence tests, alongside ultrasoft pseudopotentials [57] and a self-consistent field (SCF) tolerance of 1.0 × 10^−5^ eV/atom. This cutoff of 450 eV was selected as it provides an optimal balance between computational efficiency and accuracy, ensuring convergence of the total energy to within 1 meV/atom. The SCF tolerance and geometry optimization criteria are standard, rigorous thresholds for obtaining reliable electronic structures and relaxed geometries in solid-state systems. Brillouin zone sampling utilized a 2 × 2 × 1 k-point grid for the bulk unit cell [58] and the Γ-point for the larger surface models (supercells: ~200 atoms), with tests confirming a negligible impact on adsorption energies (<0.01 eV) [58,59].

The kaolinite (001; aluminol) and (00-1; siloxane) basal surfaces were modeled using a standard single-layer 3 × 2 × 1 supercell [45,60]. A vacuum spacing of 20 Å along the z-axis prevented spurious periodic interactions [61].

By cleaving the kaolinite model using the “Cleave Surface” function of the software, resulting in (001) and (00-1) as the predominant crystallographic. The (001) surface is characterized by an Al–OH termination, whereas the (00-1) surface is terminated by a Si–O (siloxane) layer. A comparative analysis of adsorption energies between the (001) and (00-1) planes was performed using pristine periodic slab models; consequently, surface defects and explicit edge sites were excluded from this investigation.

The physical realism of the slab models was confirmed by calculating the surface energy (γ) using the following equation:

(1) $$\gamma \;=\;\frac{E_{\mathrm{slab}}-N\times E_{\mathrm{bulk}}}{2A}$$

where *E_slab_* is the total energy of the relaxed slab, *E_bulk_* is the energy per bulk formula unit, *N* is the number of formula units in the slab, and *A* is the surface area. The calculated surface energies of 289 mJ m^−2^ (001) and 297 mJ m^−2^ (00-1) agree with established benchmarks [62]. Representative edge terminations with protonated/deprotonated groups [18,63] and organically modified aluminol surfaces [64,65] were also constructed to assess a broader range of surface chemistries.

The isolated quercetin molecule was optimized in a large orthorhombic box (*V* ≈ 1721.6 Å^3^) to prevent intermolecular interactions. For electronic structure analysis (HOMO-LUMO, Mulliken charges, Fukui indices), calculations were performed with the Dmol^3^ module using a GGA-PBE functional and a DNP 4.4 basis set.

### 2.3. Monte Carlo Simulations (MC)

**Figure 2:**
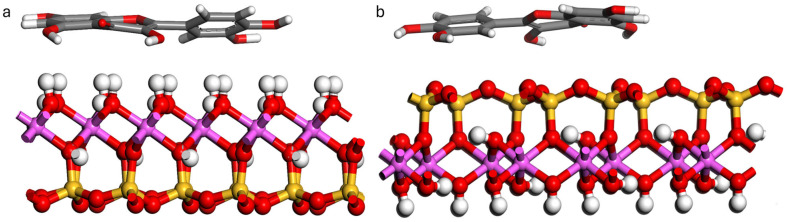
Initial configurations of quercetin adsorbed on kaolinite (**a**) (001) and (**b**) (00-1) surfaces. Atoms of Al, Si, H, C and O are pink, yellow, white, gray and red.

To locate low-energy adsorption sites on the adsorbent surfaces, MC generated initial adsorption configurations with the Adsorption Locator module of Materials Studio 8.0 software [66]. Adsorption behavior was modeled using the Universal Force Field (UFF) [67]. The choice of UFF stems from its notable transferability and performance; it has been validated against DFT with an energy drift of less than 5%, indicating its reliability for initial configurations. van der Waals interactions were described with the atom-based summation method [68]. The Ewald summation method was adopted to calculate the electrostatic force [69]. The calculations were performed at the Ultrafine quality level using simulated annealing with 10 cycles of 100,000 steps each (1,000,000 steps in total), with geometry optimization enabled and an automated temperature schedule. At the end of these cycles, the initial configurations of quercetin on kaolinite (001) and (00-1) surfaces were obtained (Figure 2).

Sampling convergence was evaluated by monitoring the running minimum adsorption energy over annealing cycles and confirming that no further decrease occurred.

The MC simulations were performed using the basal slabs of kaolinite (001) (aluminol) and (00-1) (siloxane), which were also employed in the DFT calculations.

Based on the most stable configurations identified by MC, further optimization was carried out using DFT. The chemical reactivity was analyzed using the molecule’s frontier molecular orbitals. All calculations were performed in reciprocal space.

### 2.4. Adsorption Energy Analysis

The adsorption energy (*E_ads_*) is used to assess the stability of the adsorbate on the surface. It is expressed in kJ mol^−1^ and calculated by the formula (Equation (2)):

(2) $$E_{ads}=E_{tot}-\left(E_{surf}+E_{mol}\right)$$

*E_ads_*: adsorption energy, *E_tot_*: energy of the kaolinite surface (001) or surface (00-1) containing an adsorbed quercetin molecule, *E_mol_*: energy of quercetin in a (a × b × c) Å^3^ cubic box, *E_surf_*: kaolinite surface energy (001) or (00-1).

When the adsorption energy is negative (*E_ads_* < 0), this indicates that adsorption is thermodynamically favorable, spontaneous and exothermic. The lower the *E_ads_* value, the more stable the adsorption.

### 2.5. Prospective Explicit-Solvent MD and PMF Protocol

The prospective protocol for computing the adsorption and desorption free energy profiles of quercetin on kaolinite in aqueous and hydroethanolic solutions involves classical molecular dynamics (MD) simulations with explicit solvent. Sampling is conducted along the collective variable (CV) *z*, defined as the distance between the adsorbate’s center of mass and the material surface, using the umbrella sampling method with successive windows of width Δ*z* ≈ 0.25 Å, spanning the range from the surface to the bulk solvent [21,38]. The potential of mean force (PMF, or *W*(*z*)) is reconstructed from the biased histograms using the Weighted Histogram Analysis Method (WHAM), with the free energy obtained from the fundamental relation (Equation (3)):

*W*(*z*) = −*k_B_T* *lnP*(*z*) (3)

where k_B_ is Boltzmann’s constant, T is the temperature, and *P*(*z*) is the probability distribution along *z*.

To characterize adsorption motifs (flat, tilted, vertical) and associated energy barriers, orientational collective variables are monitored, with their sampling enhanced by a complementary well-tempered metadynamics approach [23,70]. The resulting free energy barriers are used to calculate residence times and desorption rates via Transition State Theory, enabling the investigation of kinetic temperature dependence. This explicit-solvent MD approach, which provides critical entropic and solvation contributions, complements dispersion-inclusive Density Functional Theory (DFT) calculations for accurately describing hybrid inorganic/organic interfaces [54]. Prior to simulations, the various protonation microstates of quercetin are accounted for, with their statistical weights in the initial sampling ensemble determined using Boltzmann factors derived from estimated solution pKa values to ensure consistency with the studied pH conditions [18]. To probe cooperative effects at finite loading, grand-canonical Monte Carlo (MC) simulations are performed to sample increasing surface coverage (θ ≈ 0.05–0.5), followed by short DFT relaxations on representative snapshots to capture aggregation, π-π stacking, and H-bond networks [23,71]. To enhance the rigor of the study, several methodological extensions are proposed. These include accounting for explicit solvation and pH-dependent speciation to model protonation microstates and cation chelation [31], as well as incorporating surface diversity (basal, edge, and organically modified) and defects or dopant [72,73,74]. Furthermore, it is essential to sample coverage effects via Monte Carlo to capture cooperative interactions [75] and to conduct cross-validation with spectroscopic or batch adsorption data [76]. Finally, the sensitivity to exchange-correlation (XC) functionals and dispersion corrections (e.g., PBE-D3 vs. nonlocal variants) is assessed, with qualitative trends remaining robust; all input structures, MC/MD scripts, and analysis workflows are shared to ensure reproducibility.

## 3. Results and Discussion

### 3.1. Optimized Kaolinite Unit Cell and Quercetin

**Figure 3:**
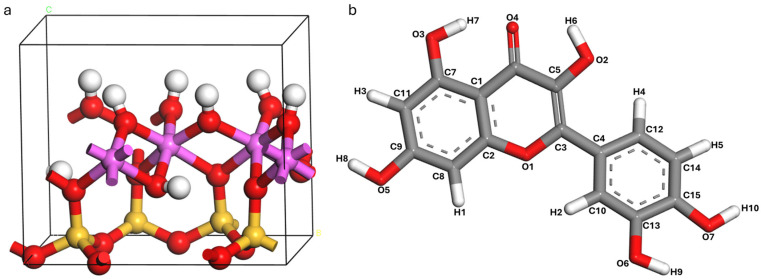
DFT-optimized of (**a**) kaolinite unit cell and (**b**) quercetin. Atoms of Al, Si, H, C and O are pink, yellow, white, gray and red.

The kaolinite unit cell, with the formula Al_4_Si_4_O_10_(OH)_8_, contains two structural units with a total of 34 atoms (Figure 3a). The hydroxylated (001) and siloxane (00-1) surfaces are the surfaces of primary interest in adsorption studies [77]. The lattice parameters of the kaolinite unit cell with the structural formula are listed in Table 1. The structural optimization of the kaolinite surface slab and quercetin adsorbate employed a rigorously validated computational approach to ensure accurate modeling of interfacial interactions. A 3 × 2 × 1 supercell was constructed from the kaolinite unit cell [78], generating a slab with a thickness of ~20 Å corresponding to more than five atomic layers, sufficient to reproduce bulk-like substrate properties while preserving surface characteristics. The lateral surface area of flat-lying quercetin (2D footprint), estimated at 90–120 Å^2^ [42,54], provides significant advantages for adsorption studies. It readily accommodates the quercetin molecule, measures around 12 Å in length and 5 Å in width [79], a representative flavonoid structure, while maintaining a critical minimum buffer distance of 5.0 Å between periodic adsorbate replicas in the *xy*-plane. This spatial separation is essential to mitigate artificial interactions imposed by periodic boundary conditions. Crucially, full atomic relaxation of all slab components, including subsurface layers, was performed during optimization to account for substrate flexibility, thereby preventing unphysical constraints that could compromise adsorption energy calculations and interfacial geometry. The configuration of the optimized quercetin molecule is shown in Figure 3b. With formula C_15_H_10_O_7_, it contains 7 oxygen atoms, 15 carbon atoms and 10 hydrogen atoms.

### 3.2. Calculation of Frontier Orbitals

**Figure 4:**
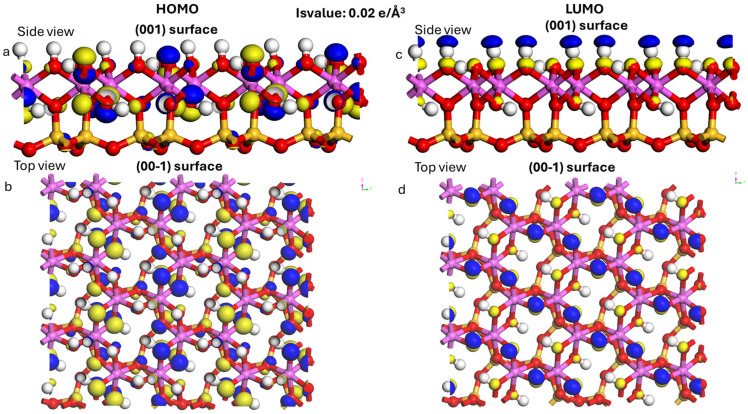
The frontier orbitals: (**a**) Kaolinite HOMO side view, (**b**) kaolinite HOMO top view, (**c**) Kaolinite LUMO side view, (**d**) Kaolinite LUMO top view. Atoms of Al, Si, H, C and O are pink, yellow, white, gray and red. The yellow area indicates higher electron density and the blue area indicates lower electron density.

**Figure 5:**
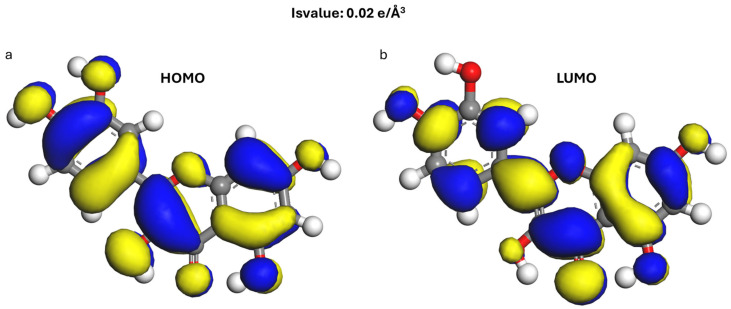
The frontier orbitals of Quercetin: (**a**) HOMO, (**b**) LUMO (The blue and yellow regions represent the orbital regions). Atoms of H, C and O are white, gray and red.

Figure 4 and Figure 5 show the distribution maps of the LUMO and HOMO frontier orbitals of kaolinite and quercetin. Fukui’s frontier molecular orbital (FMO) theory plays an interesting role in predicting chemical reactivity and revealing the reaction mechanism [84]. The HOMO orbital acts mainly as an electron donor and the LUMO as an electron acceptor [85].

**Figure 6:**
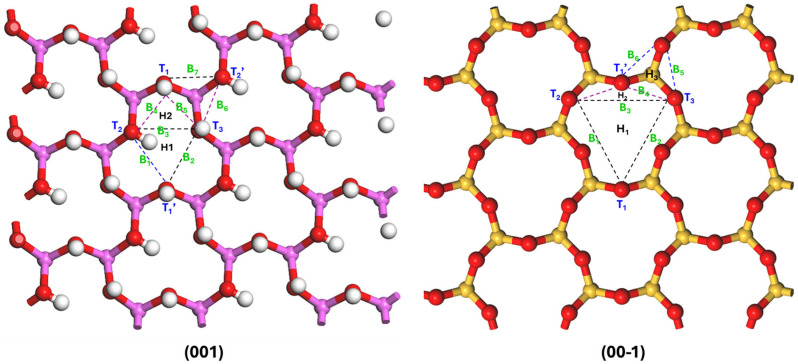
The initial adsorption sites of adsorbates on the kaolinite (001) and (00-1) surfaces. (Top sites (T), Hollow sites (H), Bridge sites (B)) [33]. Atoms of Al, Si, H and O are pink, yellow, white and red.

Based on the symmetry of the surface structure, specific sites (T, B, H) on the (001) and (00-1) kaolinite surfaces were selected as preferential sites for quercetin adsorption; T: top sites, B: bridge sites, and H: hollow sites, as shown in Figure 6 [33].

The hydroxyl and carbonyl groups of the quercetin molecule were positioned directly above the sites (T, B, H) based on the results of the frontier orbital calculations. On the surface of kaolinite (001), adsorption is mainly formed by hydrogen bonds between the hydrogen atoms in quercetin and the oxygen atoms of kaolinite, and hydrogen bonds between the oxygen atoms in quercetin and the hydrogen atoms of kaolinite. However, the adsorption of quercetin on kaolinite (00-1) surface is only formed by hydrogen bonds between hydrogen atoms in quercetin and the oxygen atoms of kaolinite [56]. For the (001) surface termination, the following adsorption sites are defined: Two hollow sites (H1, H2), Five top sites (T1, T1′, T2, T2′, T3) and Seven bridge sites (B1–B7). On the symmetrically distinct (00-1) termination, the sites include: Two hollow sites (H1, H2), Four top sites (T1, T1′, T2, T3) and Six bridge sites (B1–B6). The primed sites, T1′ and T2′, are symmetry-equivalent to T1 and T2, respectively, within the surface model. Adsorption site nomenclature and functionalities are defined as follows: Top (T) sites are centered directly above surface oxygen atoms, bridge (B) sites occupy midpoints between adjacent oxygen pairs, and hollow (H) sites reside at the centroid of oxygen triads. Quercetin adsorption exhibits site-specific interactions: its hydroxyl/carbonyl groups align preferentially with T-sites, aromatic rings engage H-sites via van der Waals interactions, while B-sites mediate bonding between functional groups and surface oxygen atoms.

The absolute value of the energy difference (Δ*E*) between the HOMO of one reactant (A) and the LUMO of another (B) is closely related to the interaction activity between them, and is calculated by the formula (Equation (4)): [86].

(4) $$∆E=\left|E_{H\left(A\right)}-E_{L\left(B\right)}\right|$$

Two types of interaction occur when the quercetin molecule is adsorbed on the kaolinite surface. They correspond to the:

Transfer of HOMO electrons from the kaolinite surface to quercetin LUMO, the energy gap is denoted Δ*E*_1_ (Equation (5)).

Electron transfer is from the HOMO of the quercetin to the LUMO of the kaolinite surface, the difference is denoted Δ*E*_2_ (Equation (6)).

(5) $${∆E}_{1}=\left|E_{H\left(kaolitine\right)}-E_{L\left(quercetine\right)}\right|$$

(6) $${∆E}_{2}=\left|E_{H\left(quercetine\right)}-E_{L\left(kaolitine\right)}\right|$$

Table 2 summarizes the frontier orbital energies and energy differences for quercetin and kaolinite. For kaolinite, the HOMO energy is −7.204 eV and the LUMO energy is −1.862 eV. For quercetin, these values are −5.102 eV for HOMO and −2.764 eV for LUMO. The calculated Δ*E*_1_ is 4.44 eV and Δ*E*_2_ is 3.24 eV. Since Δ*E*_2_ is smaller than Δ*E*_1_, it suggests that the interaction between kaolinite and quercetin is more likely to occur through the HOMO orbital of quercetin and the LUMO orbital of kaolinite.

However, Δ*E*_2_ remains relatively large (~3.24 eV; HOMO_quercetin = −5.102 eV and LUMO_kaolinite = −1.862 eV), therefore the frontier-orbital criterion should be considered qualitative and interpreted alongside Δq, DED maps, and PDOS results.

### 3.3. Mulliken Charge Distribution and Fukui Index Analysis

Mulliken charge analysis offers a qualitative overview of atomic charge distribution in molecules, facilitating the preliminary identification of regions with partial charges [92]. To understand the interaction mechanisms of quercetin with the adsorbent, we performed a dual analysis focusing on both its electrostatic properties and local chemical reactivity.

First, the distribution of atomic charges was analyzed to map the molecule’s electrostatic potential, which is crucial for identifying sites prone to non-covalent interactions like hydrogen bonding. We employed both Mulliken and Hirshfeld population analyses (Table S1). The charge distribution and Fukui indices are also summarized in Figure S1 (Supporting Information). As expected, the oxygen atoms in quercetin carry significant negative charges due to their high electronegativity. Notably, O4 exhibited the most negative charge (Mulliken: −0.589; Hirshfeld: −0.2458), highlighting it as a primary site for acting as a hydrogen bond acceptor [91,92]. These charge distributions suggest that the oxygen atoms are the main centers for electrostatic attraction to positively charged sites on the adsorbent surface.

While atomic charges provide insight into the electrostatic landscape, Fukui indices offer a more rigorous, quantum-mechanical descriptor for predicting local reactivity driven by electron transfer [93]. These indices are derived from conceptual DFT and are essential for pinpointing the most reactive atoms within a molecule. The index for nucleophilic attack, f_k_^+^, identifies the sites most susceptible to receiving electrons, whereas the index for electrophilic attack, f_k_^−^, indicates the sites most prone to donating electrons [94]. The f_k_^−^ function is related to the HOMO, pinpointing the molecule’s most nucleophilic sites. Conversely, the f_k_^+^ function is related to the LUMO, identifying the most electrophilic sites [95].

For an electrophilic attack on the molecule (where quercetin acts as an electrophile), the most reactive sites are those with the highest f_k_^+^ values. The Fukui indices are summarized in Table S1 and visualized in Figure S1 (Supporting Information). Mulliken and Hirshfeld charges reveal the most electron-rich atoms, particularly O sites, which are the primary candidates for hydrogen bond acceptance and for local dipole/polarization contributions at the kaolinite interface, although the adsorbate-surface system remains overall charge neutral. The Fukui functions (f^−^ and f^+^) provide qualitative descriptors of local electron donor and acceptor tendencies, supporting the discussion of possible electron redistribution at the interface during adsorption.

Our calculations identified C6 (0.087), C3 (0.079), and O4 (0.075) as the preferred atoms for nucleophilic attack. For a nucleophilic attack on the molecule (where quercetin acts as a nucleophile), the atoms with the highest f_k_^−^ values are the most favorable. These were identified as O2 (0.069), C5 (0.059), O3 (0.046), and C15 (0.048), making them the strongest electron-donating sites [96,97].

The results indicate that quercetin’s adsorption is governed by a combination of factors. The oxygen atoms serve as centers for electrostatic interactions and hydrogen bonding, while specific carbon and oxygen atoms, identified by the Fukui indices, act as the primary soft reactive sites for electron donation or acceptance with the adsorbent surface [98]. Furthermore, under basic conditions, deprotonation of chelating sites on quercetin increased the propensity for ion-bridged adsorption in the presence of alkaline-earth cations, partly shifting the mechanism from directional hydrogen bonding to cation-mediated coordination, in agreement with pH- and cation-dependent adsorption trends on clay minerals [18,65].

### 3.4. Analysis of Adsorption Configurations

**Figure 7:**
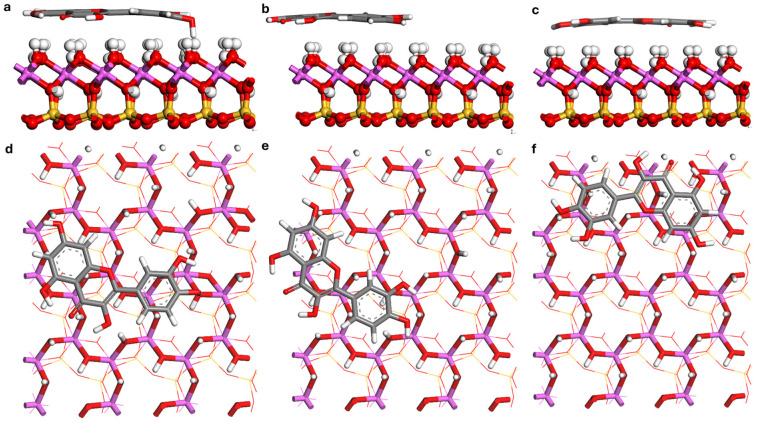
Optimized configurations of quercetin adsorbed on the kaolinite (001) surface. Side view: (**a**) Conf-001-A, (**b**) Conf-001-B, (**c**) Conf-001-C. Top view: (**d**) Conf-001-A, (**e**) Conf-001-B, (**f**) Conf-001-C. Atoms of Al, Si, H, C and O are pink, yellow, white, grey and red.

**Figure 8:**
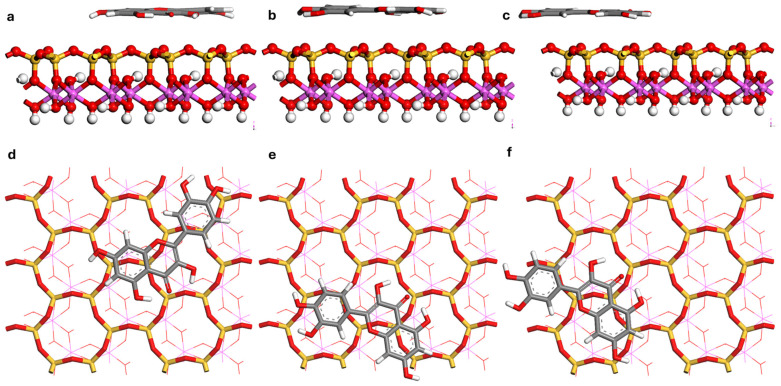
Optimized configurations of quercetin adsorbed on the kaolinite (00-1) surface. Side view: (**a**) Conf-00-1-A, (**b**) Conf-00-1-B, (**c**) Conf-00-1-C. Top view: (**d**) Conf-00-1-A, (**e**) Conf-00-1-B, (**f**) Conf-00-1-C. Atoms of Al, Si, H, C and O are pink, yellow, white, grey and red.

**Figure 9:**
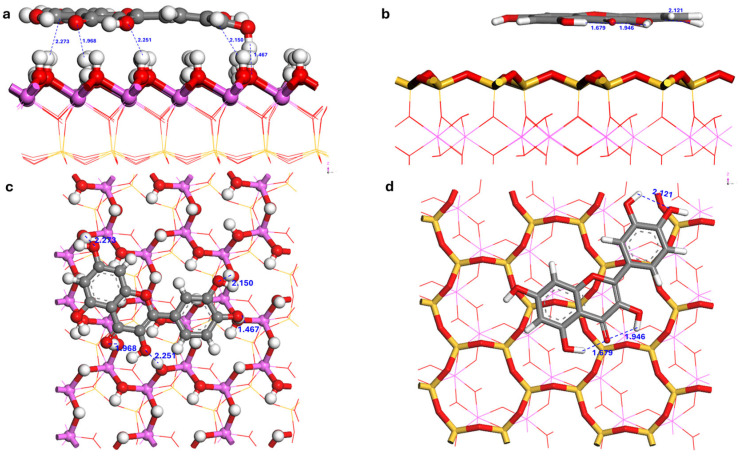
Side-by-side comparison of the most stable adsorption configurations of quercetin on kaolinite basal surfaces. Side view: (**a**) Conf-001-A, (**b**) Conf-00-1-A. Top view: (**c**) Conf-001-A, (**d**) Conf-00-1-A. Atoms of Al, Si, H, C and O are pink, yellow, white, grey and red.

Monte Carlo (MC) simulations identified multiple low-energy adsorption configurations for quercetin on the kaolinite (001) and (00-1) surfaces. The three most thermodynamically stable configurations from each surface were selected for further refinement using Density Functional Theory (DFT), with the global minimum structure depicted in Figure 7 and Figure 8. For clarity, the global minimum structures on the two basal surfaces are shown side by side in Figure 9. The calculated adsorption energies (*E_ads_*), summarized in Table 3, reveal a pronounced surface dependence. Configurations on the hydroxylated (001) surface exhibit significantly greater stability, with *E_ads_* ranging from −206.65 to −153.51 kJ mol^−1^, compared to those on the siloxane (00-1) surface, which range from −147.16 to −142.63 kJ mol^−1^. The difference of approximately 60 kJ mol^−1^ between the most stable configurations on each surface underscores a strong thermodynamic preference for the (001) face, a phenomenon attributed to cooperative hydrogen bonding. It is noted, however, that the absolute *E_ads_* values may be influenced by pH-dependent speciation effects in addition to pure binding strength [18,22]. The reported adsorption energies correspond to vacuum conditions; future work involving potential of mean force (PMF) simulations in explicit water will be necessary to determine accurate adsorption free energies (Δ*G_ads_*) and desorption barriers.

This computational result is consistent with experimental uptake data, where the adsorption capacity of kaolinite powder (*Q_max_* = 0.14 g g^−1^) is lower than that of purely hydroxylated alumina (*Q_max_* = 0.18 g g^−1^) [99], as the former represents an average over both the highly active (001) and less active (00-1) surfaces. Furthermore, the analysis of edge terminations identified additional high-affinity sites mediated by aluminol/silanol groups and hydrated microdomains. The reactivity of these edge sites, which governs pH-dependent charge development [17,24], bridges the affinity gap between the two basal surfaces and is consistent with the known face selectivity of kaolinite [18,29].

The pronounced adsorption energy calculated for the (001) surface is consistent with experimental observations confirming kaolinite’s capacity to adsorb quercetin [99] reported a maximum adsorption capacity of Q_max_ = 0.14 g g^−1^ and a Langmuir constant of K_ads_ = 46 mL g^−1^ for quercetin onto kaolinite, with a loading efficiency of 1.53 mass% determined by thermogravimetry. Furthermore, the strong interfacial interaction suggested by our DFT calculations is supported by their observed decrease in the thermal degradation temperature of adsorbed quercetin (from 353.8 °C for pure quercetin to 320.0 °C for the kaolinite hybrid), indicating a significant stabilization effect imparted by the clay surface. The prominence of hydrogen bonding as the stabilizing mechanism on the hydroxylated surface, as identified by DFT, aligns with the fundamental understanding of kaolinite’s amphoteric site chemistry [17] and is mirrored in the adsorption behavior of other organic compounds, such as carbaryl, where H-bonding is a dominant mechanism [22].

The magnitude of the most stable computed adsorption energies for quercetin on kaolinite (down to about −206.65 kJ mol^−1^) falls within the range of strong surface complexation, being comparable to first-principles binding energies reported for hydrated Pb(OH)^+^ complexes on the same basal surface [38]. This underscores that multiple H-bonds can yield strongly exergonic adsorption, a concept supported by independent experimental studies on kaolinite. For instance, the pH-dependent adsorption of glycine, with maximal uptake in conditions favoring H-bonding [18], and the control of adsorption by the protonation state of distinct Si- and Al-terminated sites [17]. All reinforce the mechanistic drivers of H-bonding and surface coordination inferred from our calculations.

Analysis of the optimized structures (Figure 7, Figure 8 and Figure 9) and bond lengths (Table 4) reveals distinct, polarity-governed adsorption mechanisms for quercetin on kaolinite surfaces. On the polar octahedral (001) surface, quercetin adopts a near-parallel orientation, stabilized by five interfacial hydrogen bonds. These consist of two short bonds (H10—O_s_50: 1.467 Å; O4—H6: 1.968 Å) and three of moderate length (O2—H11: 2.150 Å; O5—H27: 2.251 Å; O6—H42: 2.273 Å). Here, the carbonyl group (O4) acts as a hydrogen bond acceptor, while the molecule’s hydroxyl groups (O2, O5, O6) serve as proton donors to surface oxygen atoms. This directional bonding results in a strong interaction with an adsorption energy of −206.65 kJ mol^−1^ and a short average adsorbate-surface separation of 2.0–2.3 Å.

In contrast, adsorption on the non-polar tetrahedral (00-1) surface is also parallel but involves no direct hydrogen bonds with the surface. The interaction is mediated solely by non-directional van der Waals (vdW) and hydrophobic forces, leading to a weaker binding and a larger separation distance of 3.2–3.5 Å. It is important to note that the H-bond distances of 1.68–2.12 Å listed for the Quer/kaolinite (00-1) system in Table 5 correspond to intramolecular hydrogen bonds within the quercetin molecule itself, not interfacial bonds with the surface. In such systems, nonlocal vdW contributions can account for 50–60% of the attractive interactions, qualitatively reshaping the adsorption potential-energy landscape [38,54].

This pronounced selectivity quantitatively confirms the preferential adsorption on the (001) surface, driven by its surface electronic density and local aluminol hydroxyl density, which steer electron donation/acceptance and the configurational entropy landscape [21,34]. This work extends beyond prior qualitative suggestions by precisely quantifying the critical role of the surface aluminol groups in the adsorption mechanism.

### 3.5. Mulliken Bond Populations Analysis

Mulliken population analysis was performed on the DFT-optimized structures of the most stable adsorption configurations (Conf-001-A and Conf-00-1-A) to directly identify and quantify the nature of intermolecular interactions, particularly hydrogen bonding, between quercetin and the kaolinite surfaces. The results, summarized in Table 4, provide quantitative evidence for distinct adsorption mechanisms governed by surface chemistry.

On the hydroxylated (001) surface, the analysis confirms the formation of five intermolecular hydrogen bonds, which rationalizes the high adsorption energy (*E_ads_* = −206.65 kJ mol^−1^). These bonds can be categorized by length and population into two strong bonds (H10—O_s_50: 1.467 Å, pop. 0.21; O4—H_s_6: 1.968 Å, pop. 0.03) and three moderate strengths (O2—H_s_11: 2.150 Å; O5—H_s_27: 2.251 Å; O6—H_s_42: 2.273 Å). This classification aligns with established criteria where strong hydrogen bonds feature H—O distances < 2.14 Å, while medium-to-weak bonds fall within 2.14–2.56 Å, with bond populations becoming negligible beyond 3.0 Å [100]. As shown in Figure 7a,d, quercetin adopts a near-parallel orientation, maximizing surface contact. Its carbonyl group (O4) functions as a hydrogen bond acceptor from a surface aluminol group (H_s_6), while its hydroxyl groups (O2, O5, O6) act as proton donors to surface oxygen atoms.

In contrast, Mulliken analysis for the (00-1) surface confirms the absence of intermolecular hydrogen bonds between quercetin and the surface (Figure 8). The observed H—O distances for this system arise exclusively from intramolecular hydrogen bonds within the quercetin molecule; no interfacial quercetin-siloxane hydrogen bonding was identified. The adsorption is therefore mediated solely by non-directional van der Waals forces and hydrophobic effects, a direct consequence of the siloxane surface’s lack of proton donors. The hydrogen bonds listed in Table 4 for this system are exclusively intramolecular, stabilizing a specific conformation of the polyphenolic compound but not contributing to surface adhesion.

The strength of the hydrogen bond between quercetin’s H10 atom and the surface O_s_50 atom (1.467 Å) on the (001) surface is particularly notable. This surface-resolved adsorption energy is consistent with fundamental determinants of interfacial bonding, where adsorbate valence/electronegativity and the coordination environment of surface atoms collectively control bond strength [101]. The (001) aluminol surface provides a high density of hydrogen-bond donor/acceptor sites, creating local coordination anisotropy that strengthens the interaction relative to the homogeneous, non-reactive (00-1) siloxane face.

The calculated adsorption energies for all configurations are negative (ranging from −206.65 to −153.51 kJ mol^−1^ on (001) and −147.16 to −142.63 kJ mol^−1^ on (00-1)), confirming exothermic adsorption. The significant energy difference of approximately 60 kJ mol^−1^ between the most stable configurations on each surface underscores the profound impact of surface chemistry, directly attributable to the cooperative hydrogen bonding on the hydroxylated face. Furthermore, at increasing coverage, lateral quercetin–quercetin π-π and H-bond networks stabilized multi-molecule domains, partially compensating for the lack of surface H-bonds on (00-1), qualitatively altering the adsorption isotherm shape, as seen for other aromatics on minerals [22,71]. The adsorption energy (E_ads_) provides a direct measure of the enthalpy change (ΔH) for the process, which is strongly exothermic. However, the thermodynamic spontaneity of adsorption is governed by the Gibbs free energy (ΔG = ΔH − TΔS). Adsorption inherently confines molecular motion, leading to a significant loss of entropy (ΔS < 0). The dominance of specific hydrogen bonding on the (001) surface aligns with the known sensitivity of adsorption thermodynamics to surface hydroxyl density and the associated entropic penalties at oxide interfaces, particularly at low coverages [34]. For the (001) surface, the substantial exothermicity (ΔH ≈ −207 kJ mol^−1^) is the dominant term in the ΔG expression, overriding the entropic contribution and ensuring a spontaneous process (ΔG < 0). This indicates robust and stable interfacial interaction, projected to persist across ambient and elevated temperatures, a critical attribute for practical kaolinite-based nanocarrier applications. Nevertheless, a conclusion based solely on the dominance of exothermic E_ads_ over ΔG must be treated with caution, as it inherently overlooks entropic effects and competitive solvent displacement [102,103]. Therefore, a quantitative determination of the adsorption free energy (ΔG_ads_) via the potential of mean force (PMF) in explicit solvent remains an essential objective for future work.

Mulliken population analysis quantitatively demonstrates a polarity-controlled adsorption switch. The hydroxylated (001) surface facilitates strong, multi-site hydrogen bonding, leading to high-affinity, oriented adsorption. The siloxane (00-1) surface, incapable of hydrogen bonding, yields weaker, non-specific adsorption driven solely by van der Waals interactions. It is important to note that the current study focuses on pristine kaolinite surfaces in vacuo. Future work should incorporate explicit solvation, pH effects, and surface defects to better approximate experimental conditions. Competitive adsorption with water, pH-dependent deprotonation of quercetin, and the presence of edge sites or dopants could significantly modulate the adsorption behavior [27,31,32]. This mechanistic understanding, supported by the significant adsorption energy difference, provides a robust foundation for designing kaolinite-based nanocarriers where surface selection can dictate quercetin loading and release kinetics.

### 3.6. Differential Electron Density Analysis (DED)

**Figure 10:**
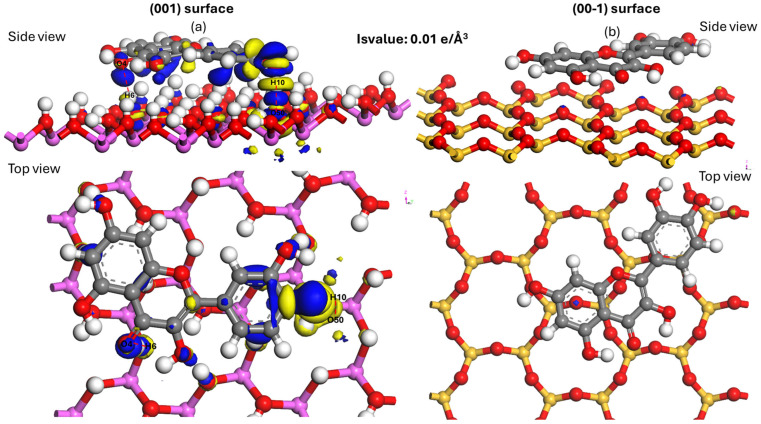
Electron density difference map of the adsorption configurations of quercetin on kaolinite (**a**): (001) (Conf-001-A) and (**b**): (00-1) surface (Conf-00-1-A). (The isosurface values in (**a**,**b**) are 0.01 electrons/Å^3^). Atoms of Al, Si, H, C and O are pink, yellow, white, grey and red.

To visualize the electronic rearrangement resulting from the interaction in the most energetically favorable system, Electron density difference maps revealed significant electron redistribution only on the Conf-001-A configuration (Figure 10). On (00-1), negligible electron transfer was observed, aligning with weak van der Waals interactions. Under explicit hydration, the first water layer outcompeted quercetin at the siloxane face, consistent with preferential water adsorption and field-enhanced stabilization of interfacial water films that suppress hydrophobe binding at (00-1) [23,70]. These patterns were consistent across all top DFT-optimized configurations. This analysis illustrates the charge transfers that stabilize the adsorption within the validated ground-state structure. The electron density difference allows for the visualization of electron redistribution and displacement of the atoms involved in quercetin binding on both kaolinite surfaces. The blue region indicates electron accumulation or enrichment, while the yellow region represents a depletion of electron density. Furthermore, the region of electron accumulation or depletion for hydrogen atoms (Hm) of the adsorbed molecule and surface oxygen (O_s_n) of kaolinite is greater than that observed for oxygen atoms (Om) of the adsorbed molecule and surface hydrogen (H_s_n). We suggest that the CHm—O_s_n bond is stronger than the O—H_s_n—Om bond.

From Figure 10a, we can see that the blue and yellow areas are mainly located around the bonded atoms. This confirms electron transfer or displacement between quercetin and the kaolinite (001) surface. In addition to electron accumulation at the quercetin O atom, electron depletion is evident at the surface H atom. The blue area is larger between the quercetin H10 atom and the O_s_50 atom of the kaolinite (001) surface, indicating a greater electron transfer or displacement resulting in a stronger O_s_50—H10 bond. In this context, the displacement of electrons from the kaolinite O_s_50 atom to the quercetin H10 atom contributes to the strengthening of this interaction.

In contrast, another well-defined blue zone is observed between the quercetin O4 atom and the H_s_6 atom of the kaolinite surface, suggesting a significant electron transfer, albeit slightly less than that observed for the O_s_50—H10 bond. With a distance of 1.97 Å, this interaction remains strong, suggesting that the O4—H_s_6 hydrogen bond also plays a key role in stabilizing quercetin adsorption on the kaolinite surface (001).

Figure 10b shows the absence of blue and yellow zones between quercetin and the kaolinite surface (00-1), indicating negligible electron transfer between the two. It is suggested that quercetin and the silicate surface of kaolinite (00-1) have limited interaction due to the low electron redistribution. Combining the results of adsorption energy and Mulliken bond population calculations, it appears that quercetin adsorbs to the kaolinite (001) surface through a combination of hydrogen bonding and van der Waals attraction interactions. Thus, based on the above research results, it can be concluded that the structural difference between the kaolinite surface (001) and the surface (00-1) is the main reason for the obvious difference in their interaction with quercetin, which may lead to preferential or selective adsorption of quercetin on the surface (001). The combination of Mulliken bond population, significant charge transfer, and clear electron density redistribution provides conclusive evidence for the formation of specific hydrogen bonds on the (001) surface and their absence on the (00-1) surface.

### 3.7. Charge Transfer Analysis

To quantify the charge redistribution observed in the density difference analysis, a Mulliken charge analysis was conducted on the atoms involved in the main interactions of the global minimum configuration (Conf-001-A). Charge transfer (delocalization) between interacting atoms is an indicator of the strength of the formed bonds, particularly hydrogen bonds. The Mulliken charge populations for this optimal system are presented in Table 5.

The 1s orbital of the H10 atom gained 0.13 electrons (e), while the positive charge on the H1 atom decreased from +0.55 e to +0.43 e, indicating electron transfer from H1 to O_s_50. For the O_s_50 atom, the 2s orbital electron population remained constant at 1.85 e, while the 2p orbital lost 0.091 e. The overall charge of O_s_50 decreased from −1.1 e to −0.96 e, consistent with electron gain from H1. This interaction, confirmed by a high bond population (0.21) and short bond length (1.467 Å), shows that O_s_50 is a strong nucleophilic site, strongly attracting electrophilic H10, which enhances quercetin adsorption.

The H_s_6 atom has gained 0.03 e, and the positive charge of H_s_6 has decreased from 0.45 e to 0.42 e, indicating that it has slightly ceded electrons to O4. The O4 atom remains the same 1.83 e charge in the 2s orbital, meaning that this sublayer does not actively participate in charge transfer. Moreover, losing 0.01 e of charge in the 2p orbital, O4’s charge has gone from −0.600 e to −0.610 e. This interaction is weaker than H10—O_s_50 but is reinforced by the strongly electronegative nature of the carbonyl group (=O4), which captures a small amount of electrons from H_s_6.

The slight charge delocalization in the 2p orbital of O4 shows that this hydrogen bond is influenced by the π interactions of the carbonyl group. The number of charges transferred during the formation of the H10—O_s_50 bond was higher than during the formation of the O4—H_s_6 bond, which also indicates that the strength of the H10—O_s_50 hydrogen bond was stronger than that of the O4—H_s_6 bond.

After adsorption, interactions between quercetin and the surface (001) intensified, and two hydrogen bonds were formed between quercetin adsorbed to the surface, of which the one between the surface oxygen and hydrogen 10 of the adsorbed molecule was the stronger.

These observations confirm that hydrogen bonds between H10 and O_s_50 is more robust than that between H_s_6 and O4, in line with previous results.

The Mulliken charge analysis conducted before and after the adsorption of quercetin on the kaolinite (00-1) surface (Table 6) reveals minimal alterations, indicating a weak interaction between the kaolinite surface atoms and quercetin. This observation is consistent with existing literature, which suggests that the interaction between kaolinite siloxane surfaces and organic molecules is largely dominated by weak hydrogen bonding and electrostatic forces, resulting in limited electron density [100,104].

### 3.8. Total Charge-Transfer

The nature of the adsorbate-surface interaction was further elucidated by quantifying the total charge transfer, determined from the summation of Mulliken charge differences on the quercetin molecule before and after adsorption in its most stable configuration on each kaolinite surface. A negative value signifies a net electron transfer from the quercetin molecule to the kaolinite surface. As summarized in Table 7, adsorption results in a net electron depletion from quercetin to both surfaces. However, a significantly enhanced charge transfer is observed for the (001) facet.

Given the anomalously high Mulliken charge transfer, we systematically compared it with alternative population analysis schemes (Hirshfeld, Bader), which yielded significantly lower and more physically reasonable values; consequently, our interpretation is based on these more robust descriptors. The markedly greater charge transfer to the (001) surface (−0.198 e) compared to the (00-1) surface (−0.083 e) directly explains the significantly stronger hydrogen bonding and the more exothermic adsorption energy, thereby validating the findings from our conformational search. This pronounced electron donation to the (001) surface indicates that adsorption is governed by dominant electrostatic and hydrogen-bonding interactions, whereas the minimal charge transfer associated with the (00-1) surface is consistent with a van der Waals-dominated binding mechanism.

It is critical to emphasize that the current DFT investigation models adsorption at the 0 K electronic-structure level, employing periodic slab models in a vacuum. To quantitatively assess the influence of solvation, molecular dynamics, and finite temperature on the adsorption free energy, we are currently implementing all-atom explicit-solvent molecular dynamics (MD) simulations with umbrella sampling. This forthcoming work will enable the calculation of the potential of mean force (PMF) along the surface-normal reaction coordinate (z) for the approach of quercetin towards the (001) and (00-1) surfaces in both pure water and water/ethanol mixtures (Equation (3)).

This will yield adsorption free energies and reveal preferred orientations under realistic solvation conditions [18,105]. PMFs in water quantified Δ*G_ads_* differences between faces and resolved orientation-dependent barriers; extracted desorption free-energy barriers translated into Arrhenius-like desorption rates with lower residence times on (00-1) than on (001), consistent with physisorption dominated by water competition at the siloxane surface [23,70]. The investigation of interfacial electron-density redistribution and ion-specific effects observed at solid–liquid interfaces motivates this extension and will allow us to test how surface hydroxyl density and the local electronic environment regulate adsorption geometry and functionally relevant orientations [21,104,106].

The strength and nature of quercetin’s interactions with the two kaolinite basal surfaces provide molecular-level insight into its drug release behavior. On the hydroxylated (001) surface, the molecule forms five hydrogen bonds, two strong (O—H—O, 1.72–1.76 Å) and three moderate strength (1.90–2.15 Å), resulting in a highly exothermic adsorption energy of −206.65 kJ mol^−1^. This strong binding is correlated with a substantial charge transfer of −0.198 e, indicative of significant electronic delocalization between the adsorbate and the surface. However, it is critical to acknowledge that release kinetics derived solely from the magnitude of ∣*E_ads_*∣ neglect crucial entropic and solvation contributions. In aqueous environments, the desorption process is ultimately governed by the reorganization of interfacial water layers and the local hydrophobicity/hydrophilicity of the basal face [103]. Consequently, the release rate constant (*k_rel_*) is more accurately described by transition state theory, as expressed in Equation (7):

(7) $$k_{rel}=A\;e^{-∆G_{rel}/RT}$$

where Δ*G_rel_* is the free energy required to disrupt the adsorbed state.

Because Δ*G_rel_* is directly proportional to the adsorption energy and the cumulative strength of hydrogen bonds, the (001) surface exhibits much slower release kinetics compared to the (00-1) surface. For instance, assuming an attempt frequency (A) of 10^12^ s^−1^ and an adsorption energy difference of 65.7 kJ mol^−1^ between the two surfaces, the model predicts a ~10^5^-fold lower desorption rate for quercetin bound to the hydroxylated surface under physiological conditions (310 K).

In contrast, on the siloxane (00-1) surface, quercetin interacts only through weak van der Waals forces (no hydrogen bonds), yielding a lower adsorption energy of −147.16 kJ mol^−1^ and a minimal charge transfer of −0.083 e. This weaker interaction corresponds to a much lower Δ*G_rel_*, enabling faster release of quercetin from this surface. The correlation between charge transfer and retention suggests that charge delocalization may serve as a comparative descriptor within the present data set: the hydroxylated (001) surface, which exhibits a larger |Δq|, stronger hydrogen bonding, and more exothermic adsorption, is expected to promote higher retention than the siloxane (00-1) surface.

In this work, Δq is used only as an internal comparative descriptor (identical computational protocol and analysis) and is interpreted alongside complementary indicators, including DED maps and PDOS. Within this consistent framework, the larger magnitude of charge transfer on the (001) surface compared with (00-1) correlates with stronger interfacial hydrogen-bonding motifs and more exothermic adsorption energy, suggesting higher retention on (001) in this system.

These quantitative relationships establish a direct link between the molecular-scale adsorption phenomena revealed in our simulations and the macroscopic release profiles observed experimentally. Consequently, surface polarity not only dictates the loading efficiency of kaolinite nanocarriers but also offers a tunable parameter for designing controlled-release systems by selectively engineering surface exposure and environmental triggers such as pH or solvent polarity, Table 8.

### 3.9. Electronic Structure and Surface Polarity-Guided Adsorption Mechanism

**Figure 11:**
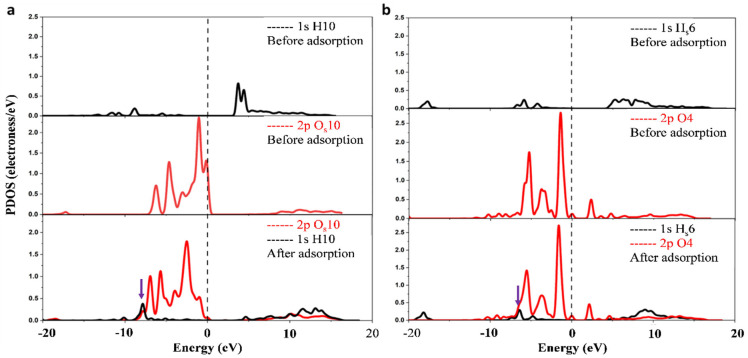
Comparative PDOS plots for the interacting atom pairs between Quercetin and the kaolinite (001) surface (Conf-001-A) before and after adsorption: (**a**) H10 and O_s_50 and (**b**) H_s_6 and O4 atoms.

To elucidate the chemical nature of the key hydrogen bonds in the most stable quercetin-kaolinite configuration (Conf-001-A), a Projected Density of States (PDOS) analysis was performed on the critical atom pairs involved in the strongest interactions: H10—O_s_50 and H_s_6—O4 (Figure 11). This analysis probes changes in orbital hybridization and electronic structure upon adsorption.

The PDOS results reveal a consistent negative shift in the energy levels of the hydrogen-bonded atoms following adsorption, indicating a significant stabilization of the system (Fermi level, *E_f_* = 0 eV). This electronic stabilization is directly aligned with the calculated negative adsorption energies, providing an electronic-structure basis for the exothermic nature of the process [107].

A detailed analysis shows distinct hybridization patterns for each bond:

For the H10—O_s_50 pair (Figure 11a), a significant overlap between the s-orbital of H10 and the p-orbitals of O_s_50 is observed near −8 eV (arrow). This substantial s-p orbital overlap is a clear signature of a strong hydrogen bond formation. Post-adsorption, the p-orbital peaks of the surface oxygen (O_s_50) vanish at the Fermi level (*E_f_*), and the peak density near E_f_ decreases markedly.

For the H_s_6—O4 pair (Figure 11b), a weaker s-p orbital overlap is identified near −6.5 eV (arrow), indicating a hydrogen bond of moderate strength compared to the H10—O_s_50 interaction. This correlates well with the longer bond length and smaller Mulliken population for this bond.

The magnitude of the s-p orbital overlap provides an electronic measure that directly correlates with the relative hydrogen bond strength (H10—O_s_50 > H_s_6—O4). Collectively, the observed electronic signatures, including the negative PDOS energy shift, the disappearance of oxygen p-states at *E_f_*, the reduced density of states near *E_f_*, and the overall shift in the DOS to lower energies, indicate a reduction in the surface energy of kaolinite (001) and an enhanced stability of the adsorption complex [25]. These electronic stabilization phenomena reinforce the critical role of interfacial electronic response, consistent with benchmarks for molecule–surface systems where van der Waals-inclusive approaches and nonlocal effects govern adsorption geometries and energetics [38,54].

The comprehensive computational analysis presented herein establishes a basic principle governing flavonoid adsorption on kaolinite surfaces: surface polarity acts as the primary determinant of adsorption strength, selectivity, and subsequent release behavior. DFT calculations reveal that the intrinsic surface polarity of kaolinite basal planes creates distinct molecular recognition environments for quercetin adsorption. The hydroxylated (001) surface, characterized by exposed Al-OH groups, presents a hydrophilic interface capable of multiple hydrogen-bonding interactions, while the siloxane-terminated (00-1) surface offers a hydrophobic environment limited to van der Waals interactions.

This surface polarity differential generates a 60 kJ mol^−1^ binding energy gradient between the two surfaces, establishing thermodynamic selectivity that can be exploited for controlled drug loading and release applications. The magnitude of this energy difference is comparable to binding energies observed in weak protein-ligand interactions, suggesting that surface polarity-mediated adsorption could provide controllable, reversible drug binding suitable for pharmaceutical applications.

Electronic analysis further reveals that surface polarity controls not only the magnitude but also the mechanism of adsorption. On the hydrophilic (001) surface, significant charge redistribution (−0.198 e) occurs through directional hydrogen bonds, creating localized electronic interactions that stabilize specific quercetin orientations. The PDOS analysis demonstrates that these interactions involve s(H)—p(O) orbital hybridization, characteristic of classical hydrogen bonding. Conversely, the hydrophobic (00-1) surface exhibits minimal charge transfer (−0.083 e), indicating that adsorption proceeds through non-directional van der Waals forces.

This fundamental difference in interaction mechanism has profound implications for drug release kinetics: hydrogen-bonded complexes on (001) surfaces will exhibit pH-dependent release profiles, while van der Waals-bound molecules on (00-1) surfaces will show primarily concentration-gradient-driven release.

## 4. Conclusions

The development of efficient carriers for the controlled release of active pharmaceutical ingredients remains a significant challenge. This study provides unprecedented atomic-level insights into the adsorption of the flavonoid quercetin onto the pristine basal surfaces of kaolinite, a naturally abundant clay mineral. Our work delivers the first direct comparative quantification of the fundamentally distinct adsorption mechanisms of quercetin on the aluminol-terminated (001) and siloxane-terminated (00-1) surfaces, offering key findings for the rational design of clay-based drug delivery systems.

The principal conclusions of this investigation are fourfold. First, we have unequivocally demonstrated a polarity-governed switch in adsorption mechanism. On the hydrophilic (001) surface, adsorption is dominated by specific hydrogen bonding, resulting in a high adsorption energy of −206.65 kJ mol^−1^.

In stark contrast, adsorption on the hydrophobic (00-1) surface is governed exclusively by non-directional van der Waals interactions, yielding a significantly weaker binding energy of −147.16 kJ mol^−1^. This energy difference of approximately 60 kJ mol^−1^ provides quantitative evidence of a strong preferential adsorption on the (001) surface, a finding distinct from studies on modified clays or smaller molecules.

Second, moving beyond phenomenological observation, we elucidated the electronic structure basis for this contrast. Detailed analyses of charge transfer, differential electron density, and bond topology fundamentally explain the selectivity. The surface aluminol groups are identified as soft reactive sites, with the intrinsic structural and electronic differences between the surfaces being the root cause of the observed affinity. The near-parallel orientation of quercetin on the (001) surface is critical for maximizing multi-site H-bond formation. These mechanistic insights align with fundamental surface science concepts, enabling predictive carrier design.

Third, the innovative integrated methodology, combining Monte Carlo (MC) and Density Functional Theory (DFT), provides a comprehensive, quantitative atomic-scale perspective, simultaneously resolving energetics, charge distribution, and bond geometries. The methodological framework, which accounts for surface diversity and can incorporate explicit hydration, is transferable to other hybrid inorganic/organic interfaces.

Finally, these findings yield actionable, application-driven insights. The pronounced preference for the (001) surface directly informs nanocarrier engineering strategies, such as the synthesis of kaolinite particles with maximized (001) surface exposure or solvent selection to promote interactions with the hydroxylated surface, ultimately aiming to improve quercetin’s bioavailability and stability in pharmaceutical, cosmetic, and food applications.

The generalizability of our approach is enhanced by its consideration of key variables, and cross-validation with simulated IR spectra supports the mechanistic model of a H-bond versus vdW-dominated switch across the crystal faces.

In summary, leveraging the superior adsorption characteristics of kaolinite’s (001) surface is a highly promising strategy for enhancing quercetin delivery. While the present DFT/MC protocol provides invaluable atomistic insights and predicts interaction sites for targeted experimental validation, this work is being actively extended.

It is important to note that these results are derived from an idealized computational model based on pristine periodic basal slabs. While this approach effectively isolates the intrinsic effects of surface polarity, real-world applications involve more complex conditions (surface defects, edge effects, full hydration). Nevertheless, the clear distinction between the hydrogen-bonding mechanism on the aluminol (001) surface and the van der Waals interaction on the siloxane (00-1) surface provides fundamental principles for the rational design of kaolinite-based nanocarriers. These intrinsic mechanisms are key to optimizing the retention and release of quercetin.

Future studies would consider molecular dynamics (MD) simulations in explicit solvent, including the calculation of the potential of mean force (PMF), to yield adsorption free energies and orientation distributions under physiologically relevant conditions, incorporating pH effects, surface defects, and competitive hydration to better align with experimental settings. Additionally, direct cross-validation with spectroscopic techniques such as FT-IR and XPS will be pursued to further strengthen the mechanistic conclusions. These ongoing efforts are crucial for bridging the gap between theoretical prediction and practical application, thereby paving the way for the rational design of optimized kaolinite-based carriers.

## Figures and Tables

**Table 1:** Lattice parameters of kaolinite unit cell.

Length (Å)			Angle (°)			References
a	B	c	A	Β	Γ	
5.1550	8.9590	7.4070	91.6800	104.9000	89.9400	[80]
5.153 (1)	8.941 (1)	7.403 (1)	91.692 (3)	104.860 (3)	89.822 (3)	[44]
5.1554 (1)	8.9448 (2)	7.4048 (2)	91.700 (2)	104.862 (1)	89.822 (1)	[81]
5.1535 (3)	8.9419 (5)	7.3906 (4)	91.926 (2)	105.046 (2)	89.797 (2)	[43]
5.196	9.009	7.372	92.996	105.979	89.877	[82]
5.154	8.942	7.391	91.93	105.05	89.80	[83]
5.1489 (9)	8.9339 (9)	7.384	91.930	105.0419 (6)	89.791	This work

**Table 2:** Frontier orbital energies and the absolute value of energy difference of the quercetin molecule and the kaolinite surface.

	E_HOMO_	E_LUMO_	Δ*E*_gap_	Δ*E*_1_	Δ*E*_2_	References
	Frontier Orbital Energy/eV			Difference/eV		
Quercetin	−5.102	−2.764	2.338	4.44	3.24	This work
	−5.930	−2.370	3.560			[87]
	−6.054	−2.094	3.960			[88]
	−6.510	−2.890	3.620			[89]
	−6.080	−2.270	3.810			[90]
Kaolinite	−7.204	−1.862	5.342	4.44	3.24	This work
	−7.309	−2.091	5.218			[91]
	−7.060	−2.130	4.930			[82]
	−7.363	−1.990	5.373			[83]

**Table 3:** DFT Adsorption Energies for Low-Energy Quercetin/Kaolinite Configurations.

Surface	Configuration ID	*E_ads_* (kJ mol^−1^)	Interaction Features
Kaolinite (001)	Conf-001-A	−206.65	Parallel orientation, 5 H-bonds (2 strong, 3 moderate)
	Conf-001-B	−183.29	Parallel orientation, 4 H-bonds (1 strong, 3 moderate)
	Conf-001-C	−153.51	Parallel orientation, 3 H-bonds (2 strong, 1 moderate)
Kaolinite (00-1)	Conf-00-1-A	−147.16	Parallel orientation, vdW and hydrophobic forces, no external H-bonds
	Conf-00-1-B	−146.17	
	Conf-00-1-C	−142.63	

**Table 4:** Mulliken bond populations and lengths for adsorbate-surface atoms in configurations Conf-001-A and Conf-00-1-A.

Adsorption System	Bond	Bond Population	Bond Lengths/Å
Quer/kaolinite (001) surface	H10—O_s_50	0.21	1.467
	O4—H_s_6	0.03	1.968
	O2—H_s_11	0.02	2.150
	O5—H_s_27	0.01	2.251
	O6—H_s_42	0.02	2.273
Quer/kaolinite (00-1) surface	H6—O4	0.13	1.679
	H7—O4	0.07	1.946
	H9—O7	0.02	2.121

**Table 5:** Mulliken charge populations before and after the quercetin adsorption on the kaolinite (001) surface (Conf-001-A).

Adsorption System	Atom	Mulliken Charge Population					
		Before			After		
		s	*p*	Charge/e	s	*p*	Charge/e
Quercetin/Kaolinite (001) surface	H10	0.445	-	0.555	0.568	-	0.432
	O1	1.735	4.647	−0.382	1.736	4.647	−0.382
	O2	1.817	4.870	−0.687	1.813	4.859	−0.672
	O3	1.806	4.858	−0.664	1.803	4.835	−0.638
	O4	1.830	4.770	−0.600	1.831	4.779	−0.610
	O5	1.818	4.863	−0.681	1.815	4.864	−0.679
	O6	1.815	4.885	−0.700	1.817	4.858	−0.675
	O7	1.811	4.912	−0.722	1.795	4.889	−0.684
	H_s_6	0.551	-	0.449	0.579	-	0.421
	H_s_11	0.545	-	0.455	0.548	-	0.452
	H_s_27	0.545	-	0.455	0.568	-	0.432
	H_s_42	0.545	-	0.455	0.565	-	0.435
	O_s_50	1.850	5.202	−1.052	1.849	5.111	−0.960

**Table 6:** Mulliken charge populations before and after the quercetin adsorption on the kaolinite (00-1) surface (Conf-00-1-A).

Adsorption System	Atom	Mulliken Charge Population					
		Before			After		
		s		*p*	s		*p*
		Charge/e			Charge/e		
Quercetin/Kaolinite (00-1) surface	H10	0.445	-	0.555	0.456	-	0.544
	O1	1.735	4.647	−0.382	1.736	4.645	−0.382
	O2	1.817	4.870	−0.687	1.814	4.863	−0.678
	O3	1.806	4.858	−0.664	1.804	4.842	−0.646
	O4	1.830	4.770	−0.600	1.830	4.772	−0.601
	O5	1.818	4.863	−0.681	1.814	4.869	−0.682
	O6	1.815	4.885	−0.700	1.811	4.873	−0.684
	O7	1.811	4.912	−0.722	1.809	4.902	−0.711

**Table 7:** Total Charge-Transfer from Quercetin to Kaolinite (001) (Conf-001-A) and (00-1) (Conf-00-1-A) Surfaces.

To Surface	Total Charge-Transfer
001 kaolinite	−0.198 e
00-1 kaolinite	−0.083 e

**Table 8:** Summary of results for the adsorption of quercetin.

Surface Type	H-Bonds Per Quercetin Molecule	Avg H-Bond Energy (kJ mol^−1^)	Total Adsorption Energy (kJ mol^−1^)	Charge Transfer (e)	Predicted Release Rate (k_rel_ s^−1^)
Hydroxylated (001)	5 (interfacial)	−15.6	−206.65	−0.198	3.2 × 10^−7^
Siloxane (00-1)	0	—	−147.16	−0.083	2.9 × 10^−2^

## Data Availability

The original contributions presented in this study are included in the article/Supplementary Materials. Further inquiries can be directed to the corresponding authors.

## Supplementary Materials

The following supporting information can be downloaded at: https://www.mdpi.com/article/10.3390/ma19020368/s1, Table S1. Mulliken and Hirshfeld atomic charges and Fukui indices of quercetin; Figure S1. Analysis of Charge Distribution and Fukui Indices for Quercetin.

## Abbreviations

BFGS	Broyden–Fletcher–Goldfarb–Shanno
CASTEP	Cambridge Serial Total Energy Package
CVs	Collective Variables
DED	Differential Electron Density
DFT	Density Functional Theory
DMol^3^	Density Functional Molecular Orbital Package
DNP	Double Numerical plus Polarization
E_ads_	Adsorption Energy
E_f_	Fermi level
FMO	Frontier Molecular Orbital
GGA	Generalized Gradient Approximation
HOMO	Highest Occupied Molecular Orbital
LUMO	Lowest Unoccupied Molecular Orbital
MC	Monte Carlo
MD	Molecular Dynamics
PBE	Perdew–Burke–Ernzerhof
PEO	Poly (ethylene oxide)
PDOS	Partial Density of States
SCF	Self-Consistent Field
TS	Tkatchenko–Scheffler
UFF	Universal Force Field
USP	Ultrasoft Pseudopotential
vdW	van der Waals
WHAM	Weighted Histogram Analysis Method
