# Supplementary material for: Polarity-Driven Selective Adsorption of Quercetin on Kaolinite: An Integrated DFT and Monte Carlo Study
Source: Materials (Basel). 2026 Jan 16;19(2):368. doi: 10.3390/ma19020368 (PMC12843195; doi:10.3390/ma19020368)
Supplement: Supplementary file 1 [file materials-19-00368-s001.zip › materials-4039334-supplementary.pdf]

Supporting Information

Polarity-Driven Selective Adsorption of Quercetin on Kaolinite: An Integrated DFT and Monte Carlo Study

Abdelilah Ayad[1*], Achraf Harrou[1,2], Abdelouahad El Himri[1], Mohamed Benali[3], Abdelouassia Dira[1], Santiago Aparicio[4,5*], Alberto Gutiérrez[4], Armand Soldera[5*], Elkhadir Gharibi[1*]

[1] Laboratory of Applied Chemistry and Environment, Faculty of Sciences, Mohammed First University, BP 717, Oujda 60000, Morocco. ayad1999abdo@gmail.com; gharibi_elkhadir@yahoo.fr; harrou201@gmail.com; elhimri.abdelouahad@gmail.com, abdelouassia.dira@ump.ac.ma

[2] Geology and Sustainable Mining Institute (GSMI), Mohammed VI Polytechnic University (UM6P), 43150, Benguerir, Morocco. Achraf.HARROU-EXT@um6p.ma

[3] TIMR (Integrated Transformations of Renewable Matter), Université de Technologie de Compiègne, ESCOM, Centre de Recherche Royallieu, CS 60319-60203 Compiègne Cedex, France. m.benali@escom.fr

[4] Department of Chemistry, University of Burgos, 09001 Burgos, Spain. sapar@ubu.es; agvega@ubu.es

[5] International Research Centre in Critical Raw Materials-ICCRAM, University of Burgos, 09001 Burgos, Spain. sapar@ubu.es

[6] Department of Chemistry, Université de Sherbrooke, Sherbrooke, Quebec J1K 2R1, Canada. armand.soldera@usherbrooke.ca

* Corresponding authors:

ayad1999abdo@gmail.com; gharibi_elkhadir@yahoo.fr; armand.soldera@usherbrooke.ca; sapar@ubu.es

Table S1: Mulliken and Hirshfeld atomic charges and Fukui indices of quercetin

Model	Atom	Mulliken charge (e)	Hirshfeld charge (e)	$F^-(r)$	$F^+(r)$
	O_1	**-0.435**	-0.0604	0.023	0.033
	O_2	**-0.491**	-0.1624	**0.069**	0.029
	O_3	**-0.505**	-0.1908	**0.046**	0.040
	O_4	**-0.589**	-0.2458	**0.041**	**0.075**
	O_5	**-0.446**	-0.1659	0.032	0.031
	O_6	**-0.471**	-0.1814	**0.04**	0.024
	O_7	**-0.495**	-0.1751	**0.043**	0.028
	C_1	-0.005	-0.0457	0.018	0.006
	C_2	0.329	0.0775	0.011	0.019
	C_3	0.276	0.0644	**0.045**	**0.079**
	C_4	0.082	-0.0121	0.019	0.004
	C_5	0.219	0.0396	**0.059**	0.025
Quercetin	C_6	0.289	0.0773	0.024	**0.087**
	C_7	0.333	0.083	0.027	0.037
	C_8	-0.226	-0.0824	**0.046**	0.028
	C_9	0.37	0.0804	0.026	0.034
	C_{10}	-0.164	-0.0554	0.025	0.040
	C_{11}	-0.229	-0.0907	0.033	0.030
	C_{12}	-0.12	-0.0456	0.036	0.031
	C_{13}	0.312	0.0583	0.032	0.022
	C_{14}	-0.136	-0.0616	0.023	0.021
	C_{15}	0.289	0.0519	0.048	**0.050**
	H_1	0.06	0.0541	0.03	0.028
	H_2	0.069	0.0412	0.025	0.025
	H_3	0.039	0.0393	0.024	0.032
	H_4	0.079	0.0358	0.027	0.027
	H_5	0.042	0.046	0.037	0.034
	H_6	0.291	0.1351	0.024	0.021
	H_7	0.318	0.1068	0.016	0.018
	H_8	0.262	0.1688	0.016	0.017
	H_9	0.279	0.1489	0.016	0.013
	H_{10}	0.274	0.1732	0.018	0.013

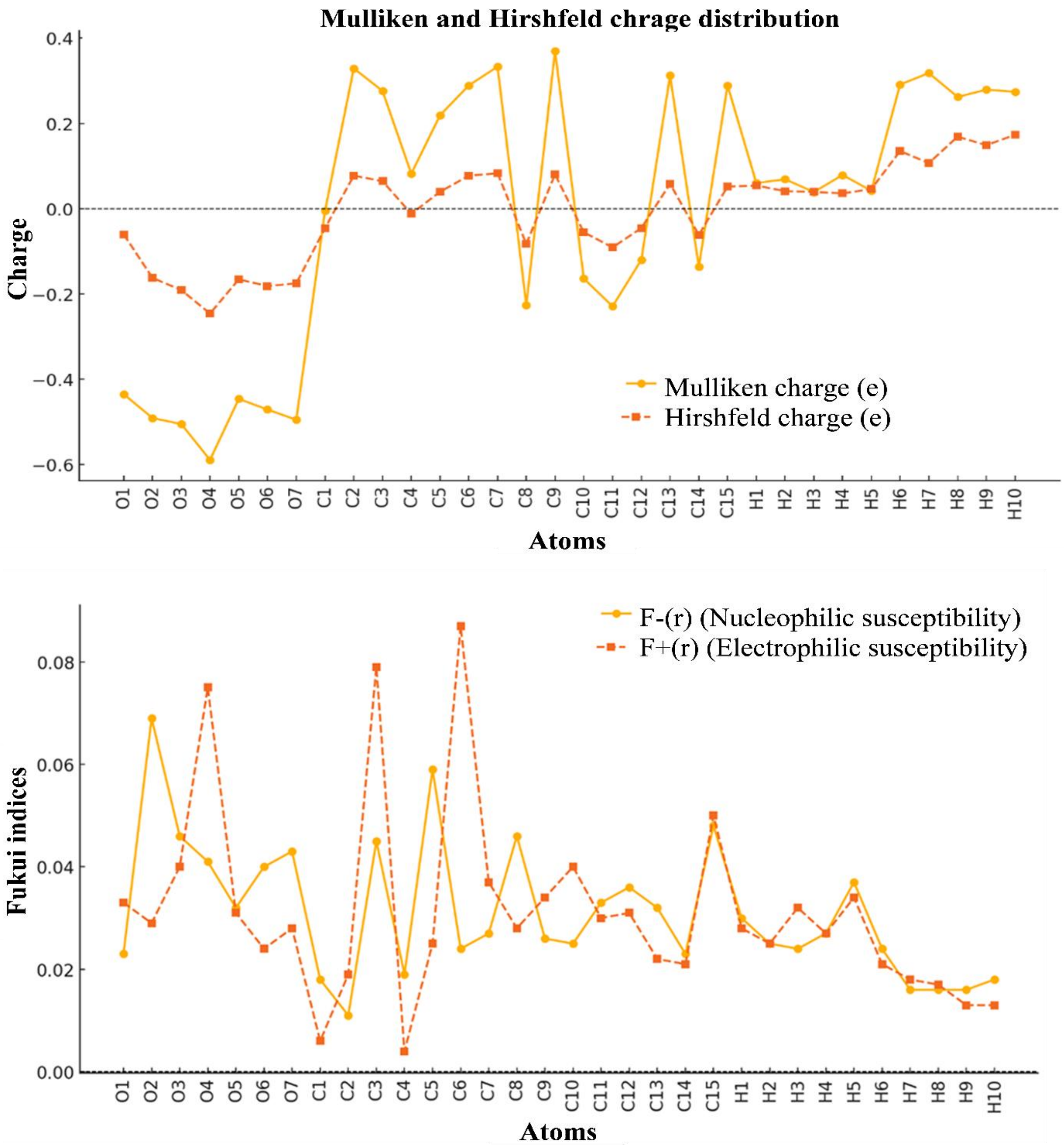

Figure S1. Analysis of Charge Distribution and Fukui Indices for Quercetin
